# Annotating Human P-Glycoprotein Bioassay Data

**DOI:** 10.1002/minf.201200059

**Published:** 2012-08-07

**Authors:** Barbara Zdrazil, Marta Pinto, Poongavanam Vasanthanathan, Antony J Williams, Linda Zander Balderud, Ola Engkvist, Christine Chichester, Anne Hersey, John P Overington, Gerhard F Ecker

**Affiliations:** a University of Vienna, Department of Medicinal Chemistry, Pharmacoinformatics Research Group, Althanstrasse 14, 1090 Vienna, Austria' phone/fax: +43-1-4277-55110/+43-1-4277-9551; b Royal Society of Chemistry, 904 Tamaras Circle, Wake Forest, NC 27587, USA; c Discovery Sciences, Computational Sciences, AstraZeneca R&D, Mölndal, Sweden; d Swiss Institute of Bioinformatics, CALIPHO Group, CMU, Rue Michel-Servet 1, 1211 Geneva 4, Switzerland; e European Molecular Biology Laboratory (EMBL)-European Bioinformatics Institute, Wellcome Trust Genome Campus, Cambridge CB10 1SD, UK

**Keywords:** P-glycoprotein, Multidrug resistance protein 1, Bioassay ontology, ChEMBL database, TP-search

## Abstract

Huge amounts of small compound bioactivity data have been entering the public domain as a consequence of open innovation initiatives. It is now the time to carefully analyse existing bioassay data and give it a systematic structure. Our study aims to annotate prominent in vitro assays used for the determination of bioactivities of human P-glycoprotein inhibitors and substrates as they are represented in the ChEMBL and TP-search open source databases. Furthermore, the ability of data, determined in different assays, to be combined with each other is explored. As a result of this study, it is suggested that for inhibitors of human P-glycoprotein it is possible to combine data coming from the same assay type, if the cell lines used are also identical and the fluorescent or radiolabeled substrate have overlapping binding sites. In addition, it demonstrates that there is a need for larger chemical diverse datasets that have been measured in a panel of different assays. This would certainly alleviate the search for other inter-correlations between bioactivity data yielded by different assay setups.

## 1 Introduction

Human P-glycoprotein (UniProt ID: P08183) is a member of the human ATP-Binding Cassette (ABC) transporter superfamily (alternative protein names: Multidrug resistance protein 1, ATP-binding cassette sub-family B member 1, P-glycoprotein 1, CD_antigen=CD243; gene names: ABCB1, MDR1, or PGY1).

It fulfils an important dual role in the process of drug discovery and development: It is recognised in its own right as a drug target but is also dreaded because of its anti-target character. Since the discovery of its role as a drug efflux pump in multidrug resistant tumour cells in 1976,^1^ there has been an on-going search for potent and selective inhibitors of P-glycoprotein, which might be used for the treatment of multidrug resistance (MDR) – one of the major obstacles for a successful cancer chemotherapy.^2^ Likewise the anti-target property of P-glycoprotein is significant, especially with respect to its importance for the pharmacokinetics of compounds being substrates of this xenobiotic efflux pump. This is of special importance at the blood-brain barrier, the gastro-intestinal tract, and in the liver.^3^

Inhibitors of P-glycoprotein can affect the disposition of co-administered substrates, thus Drug-Drug Interaction (DDI) studies need to be performed if the compound being clinically developed is known to interact with P-glycoprotein.^4^ Therefore it is of key importance to be able to study and understand the interaction of compounds with P-glycoprotein both by in silico and in vitro methods at an early phase of the drug discovery process.

On a molecular basis, P-glycoprotein is well known for its promiscuous ligand recognition pattern, paving the way for exploring the chemical world in an enquiry for new scaffolds as inhibitor leads of this protein. However, the more poly-specific P-glycoprotein was recognised to be, the more difficult it became to postulate a unique binding/transport site for ligands. This was also recently underpinned by the resolution of a mouse P-glycoprotein crystal structure, which revealed topologically distinct binding sites for ligands.^5^

Given the central importance of this drug transporter it is therefore not surprising that there is a large panel of biological assays reported in the literature probing the transport function of P-glycoprotein. However, different biological assays designed to probe the response to a certain agent will also lead to variation in the bioactivity values for a unique compound. In case of competitive inhibition of transport, the probe substrate used for measuring inhibition of transport will play a major role. In addition, the type of assay used (e.g. transport, cellular accumulation, inhibition), the cell line, and the assay conditions used (e.g. probe substrate concentration, duration of experiment) will certainly influence the outcome.

It seems per se to be a challenging task to interpret bioassay data. Also, the huge amount of bioassay data which is accessible nowadays in the world wide web demands a systematic structuring and standardization of the data point entries.^6^

With respect to small compound data the situation changed significantly when an unprecedented body of bioactivity data was made available to the public domain. This is a consequence of several factors, a change to an open innovation business model in the pharmaceutical industry, the development of public-private-partnerships such as the Innovative Medicines Initative (IMI), large screening initiatives such as the NIH Molecular Libraries and Imaging Program (MLP),^7^ and finally, large scale manual and computational indexing of the primary literature.^8^

The most prominent examples of such Open Access databases in the life sciences are the PubChem BioAssay database,^9^ and the ChEMBL database.^10^,^11^

PubChem hosts data from high throughput screening experiments (HTS), while ChEMBL is a manually curated collection of literature data, coming mainly from Structure Activity Relationships (SAR) studies. In the field of transport proteins we also would like to highlight the focussed transporter database TP-search.^12^, ^13^ Compound data presented herein was also retrieved from literature sources.

All three resources include compound bioactivity data with a brief tagline description of the underlying assays. However, the way data are retrieved and the respective output is different for each of these data sources. When the complexity of the information on the bioassays is considered, we obviously have to face some disparities while comparing data from different sources as well as comparing the individual entries internally. As databases compiled from literature sources are mainly built from Quantitative Structure Activity Relationships (QSAR) datasets. These are generally quite small and biased towards a set of structurally related compounds. Furthermore, as most of these studies are carried out in an academic set up and typically run sporadically and at low throughput, assays and assay conditions are different and often not directly comparable.

Thus, in order to establish predictive and robust in silico models covering a broad chemical space it is necessary to combine datasets from different studies. Nevertheless, the final size and quality of such benchmark datasets depends strongly on the potential of certain assays to be combined.

Being aware that expert knowledge represents a decisive skill for studying biological data, in this study we focus on human P-glycoprotein – definitely the best-characterised and bioassayed ABC-transporter so far. However, it is worth noting that the methodologies devised here for P-glycoprotein data can also be applied to data on other transporters which are, in general, less abundant.

The final aim of this study was the annotation/ classification of prominent in vitro assays used for the determination of bioactivities of human P-glycoprotein inhibitors and substrates as they are represented in the ChEMBL and TP-search Open Access databases. In addition, we explored the ability of bioassay data coming from distinct literatures sources (in ChEMBL or TP-search) to be combined with each other.

## 2 Methods

### 2.1 Regression Analysis

For creating a set suitable for QSAR studies all the data retrieved from ChEMBL (version ChEMBL_13) and TP-search (last update June 26, 2007) were grouped according to their numerical endpoints *IC*_50_ (concentration at half-maximum inhibition), *EC*_50_ (concentration at half-maximum effect), and *K*_i_ (inhibitor constant for the protein-inhibitor complex). In order to compare data sets from different assays we set a limit of at least ten compounds being measured in both assays. With this criterion, almost all the comparisons we were able to perform belong to the group ‘*IC*_50_’. For *K*_i_ readout we could only carry out one comparison, for *EC*_50_ it was not possible to find any two datasets with at least ten compounds in common.

In order to draw correlation plots and calculate the correlation coefficient *R*, the squared correlation coefficient (coefficient of determination) *R*^2^, adjusted *R*^2^, and standard error we converted the *IC*_50_ and *K*_i_ values to the p*IC*_50_ (−log*IC*_50_ [M]) and p*K*_i_ (−log*K*_i_ [M]) scale, respectively, in which higher values indicate exponentially greater potency.

In the results section *R*^2^ values are discussed. More detailed information on the regression statistics of the correlations drawn is given as Supporting Information (Table S1).

### 2.2 Creation of the Classification Dataset for Human P-Glycoprotein Inhibitors

The SDF file of chemical compounds was created using a combination of automated and manual protocols using a number of cheminformatics tools and searches of online databases. The approach included utilizing name to structure conversion tools to convert systematic names (IUPAC names generally) to chemical structures. Where necessary, stereochemistry (when defined in the systematic name) was introduced into the chemical structure manually. The software tools included ACD/Name (version 12)^14^ and the OPSIN online service.^15^ Trivial names and other synonyms were searched against public domain databases. These were generally ChemSpider,^16^ ChEBI,^17^ ChEMBL and PubChem. Results from multiple data sources were manually curated for consistency and compared to other reference resources such as the Merck Index and, where appropriate, Wikipedia. In some cases the names were ambiguous and inherent experience of the compounds under study was used to identify and include the chemicals.

## 3 Results and Discussion

### 3.1 Bioassays in ChEMBL and TP-Search for Human P-Glycoprotein

In order to get a first impression of the composition of the two databases, we determined the overlap of ChEMBL and TP-search for human P-glycoprotein inhibitors/substrates with readout *IC*_50_, *EC*_50_, and *K*_i_: out of approximately 1200 data point entries (compounds with associated bioactivities; 846 in ChEMBL and 352 in TP-search) we found only 40 compounds being present in both databases.

**Figure 1:**
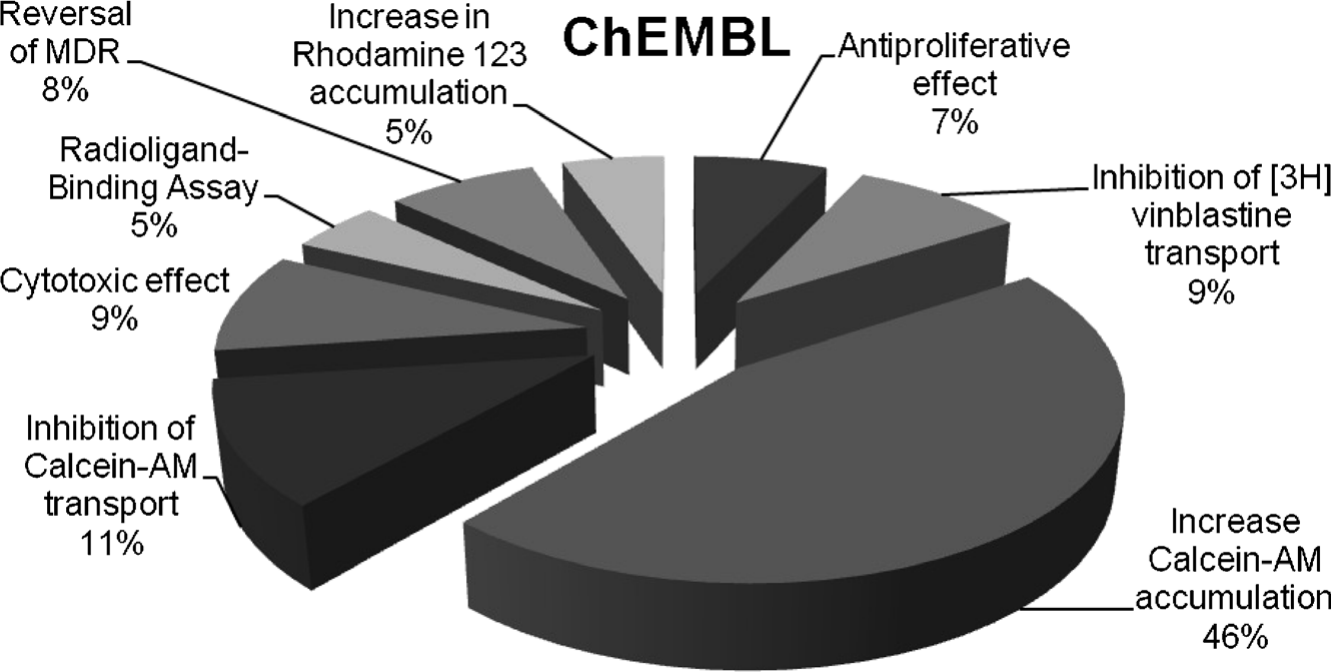
Proportion of different assays for human P-glycoprotein with readout *IC*_50_ in ChEMBL.

**Figure 2:**
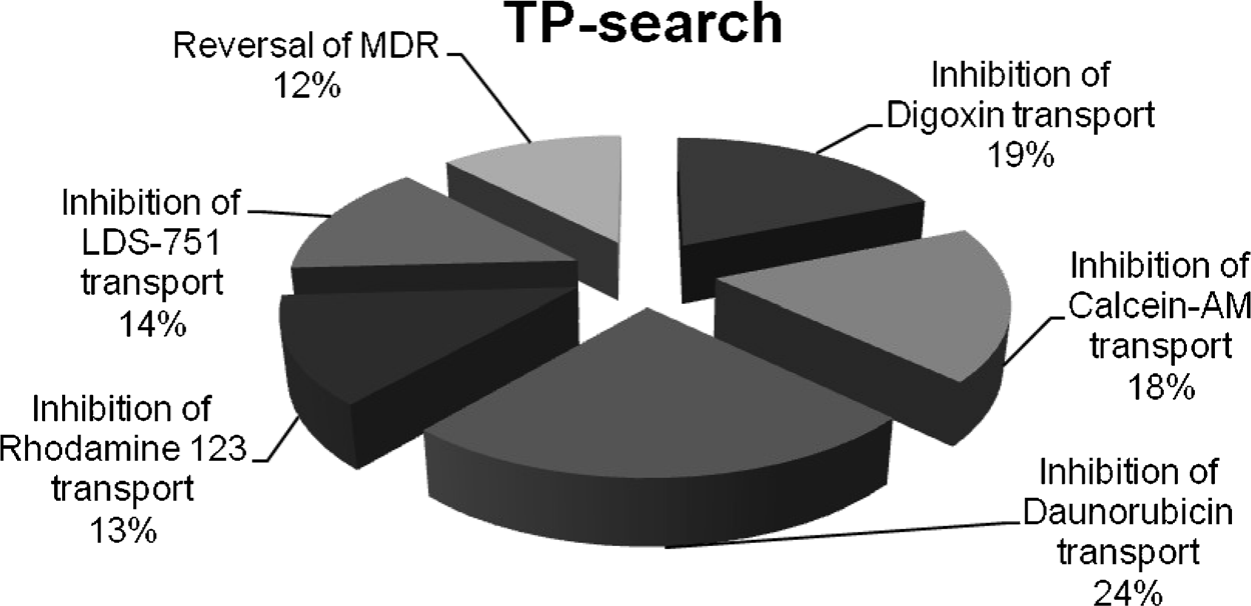
Proportion of different assays for human P-glycoprotein with readout *IC*_50_ in TPsearch.

Figure 1 and Figure 2 depict the proportion of different assays for human P-glycoprotein with readout *IC*_50_ values as they appear in ChEMBL and TP-search respectively. Figure 2 shows that the majority of assays with readout *IC*_50_ in TP-search are indirect transport assays, where the inhibition of a substrates’ transepithelial transport is measured in order to determine the inhibitory activity of the compounds under investigation. Major substrates used for these assays are daunorubicin, digoxin, calcein-AM, LDS-751, and rhodamine 123. However, in ChEMBL almost half of the assays with readout *IC*_50_ are Calcein-AM accumulation assays (where an increase in a substrates’ intracellular accumulation is measured). Five percent of the assays measure an increase in rhodamine 123 accumulation. Transport assays are underrepresented in ChEMBL when compared to TP-search (inhibition of Calcein-AM transport 11 %; inhibition of [^3^H]vinblastine transport 9 %). Other assays with an incidence of less than ten percent of all the *IC*_50_ assays in ChEMBL include the measurement of cytotoxic effects, reversal of multidrug resistance (MDR), antiproliferative effects, and radioligand-binding assays.

Inspecting the distribution of assays giving *EC*_50_ as well as *K*_i_ values in ChEMBL, we noticed that 61 % (198 compounds) of the data entries with *EC*_50_ and 73 % (80 compounds) of the data entries with K_i_ values were determined in a daunorubicin efflux (transport) assay. In TP-search there are very few data points with *EC*_50_ or *K*_i_ values so that no general tendencies of assay distributions could be deduced.

When the underlying literature sources of TP-search and ChEMBL are examined there is a clear difference in the highest contributing journal sources of data – TP-search has a higher fraction of journals concentrating on late-stage preclinical/clinical development stage reporting of a compound (e.g. Journal of Pharmacology and Experimental Therapeutics, Drug Metabolism & Disposition) whereas ChEMBL has a higher fraction of data from medicinal chemistry optimisation assays (e.g. Journal of Medicinal Chemistry, Bioorganic & Medicinal Chemistry Letters). Therefore, part of the differences in the assay distribution probably reflects custom and practice in the relevant scientific communities.

Studying the prevalence of substrates and cell lines being used in the two databases, it was observed that 45 % of all the compounds (with bioactivities in *IC*_50_, *EC*_50_ and *K*_i_) in ChEMBL are measured in assays using daunorubicin as a substrate and 29 % using calcein-AM. In TP-search there is a slightly different tendency with 24 % using calcein-AM, 17 % digoxin, and 15 % daunorubicin.

With respect to the cell lines used in the respective assays, there were around 25 different ones in each of the databases – a lot of them were identical when comparing ChEMBL and TP-search. For a list of all the cell lines used see the Supporting Information (Table S1).

Throughout our study we noticed that assay nomenclature is not homogenous. For instance, the terms ‘efflux’ and ‘transport’, which in the context of P-glycoprotein substrates are equivalent, are both used interchangeably. The same is true for the equivalents ‘uptake’ and ‘(intracellular) accumulation’. Additionally, the precision of the assay description or the assays’ name itself varies: e.g. ‘Calcein-AM efflux assay’, ‘Calcein-AM accumulation assay’, or just ‘Calcein-AM assay’, which is not even specifying if uptake or efflux is measured.

The assays were therefore first grouped according to a description of what is actually measured, which often required examining the original publications that were cited. The following major assay types were identified (X is the respective substrate used): ‘Increase in X intracellular accumulation’, ‘Inhibition of X (transepithelial) transport’; ‘Reversal of Multidrug Resistance (MDR)’; ‘Cytotoxic effect’, ‘Antiproliferative effect’, ‘Inhibition of X-stimulated ATPase activity’, and ‘Inhibition of X binding’. Further sub-classification is possible by taking into account the different substrates (X) and the cell lines used for the experiment.

From this large-scale analysis it is possible to highlight alternate expressions of the same or related assays and then develop canonical or recommended ways of expressing the assay, and driving further curation of the underlying resources.

### 3.2 Combining Bioactivities from Identical Assays

For ligand-based drug design studies it is generally highly recommended to measure all compounds in an identical assay setup. In this manner a very clean and consistent dataset is obtained (within the experimental error) which is able to reflect the structural differences in terms of differences in the pharmacological activity values.

The assembly of such a dataset (only one assay type/substrate/cell line) from a combination of ChEMBL or TP-search datasets makes it evident that the largest compound dataset that can be retrieved from ChEMBL comprises 198 entities with *EC*_50_ values measured in a daunorubicin efflux assay in MDR CCRF vcr1000 cells. The size of this dataset definitely satisfies QSAR-related studies, as does the activity range of six orders of magnitude. However, one weakness might be the lack of structural diversity of most of the compounds.^18^–^20^ From a search of TP-search we could extract a dataset of 37 compounds with *IC*_50_ values being the most comprehensive one. Compounds were measured in a daunorubicin transport assay/NIH-3T3-G185 and comprise a sufficient degree of structural diversity. Bioactivities in this dataset span three orders of magnitude.

### 3.3 Correlating Bioactivities from Different Assay Setups

In principle there are two general types of models that can be generated, those based on QSAR methods and those based on classifications. When combining data from different assays certain requirements need to be fulfilled. The combination of QSAR datasets with a significant overlap of compounds tested in both assays is highly recommended. Subsequent correlation analysis will then show if the two assays can be combined using regression analysis.

Interestingly, despite the huge number of data point entries (∼1200) at our disposal when performing the study, there were only very few datasets with a sufficient number of compounds (at least ten) available which have been tested in more than one assay. Thus, the number of correlations we were able to establish was very limited.

In the case of classification models the definition of a reference compound which is routinely tested in all assays, and used as a threshold for defining active/inactive, serves as a valuable approach. It would be a great help to the community if publications reporting frequently used assays reported explicitly such internal standard data.

For this study, it also must be clearly stated, that the subsequent analysis does not take into account assays especially designed for measuring substrates of P-glycoprotein, such as in vitro (direct) transcellular transport assays. These assays directly measure transport of substrate but report transport ratios (basolateral-to-apical vs. apical-to-basolateral) that are not as easy to correlate with each other as compared to assays based on *IC*_50_, *EC*_50_, and *K*_i_ values. Thus, our annotation scheme, and the conclusions we are drawing, are especially aimed for inhibitors of human P-glycoprotein, although this does not preclude that certain compounds discussed herein could also be classified as substrates in other assays.

#### 3.3.1 Comparison: Same Assay Type, Different Substrates (X), Same Cell Line

**Figure 3:**
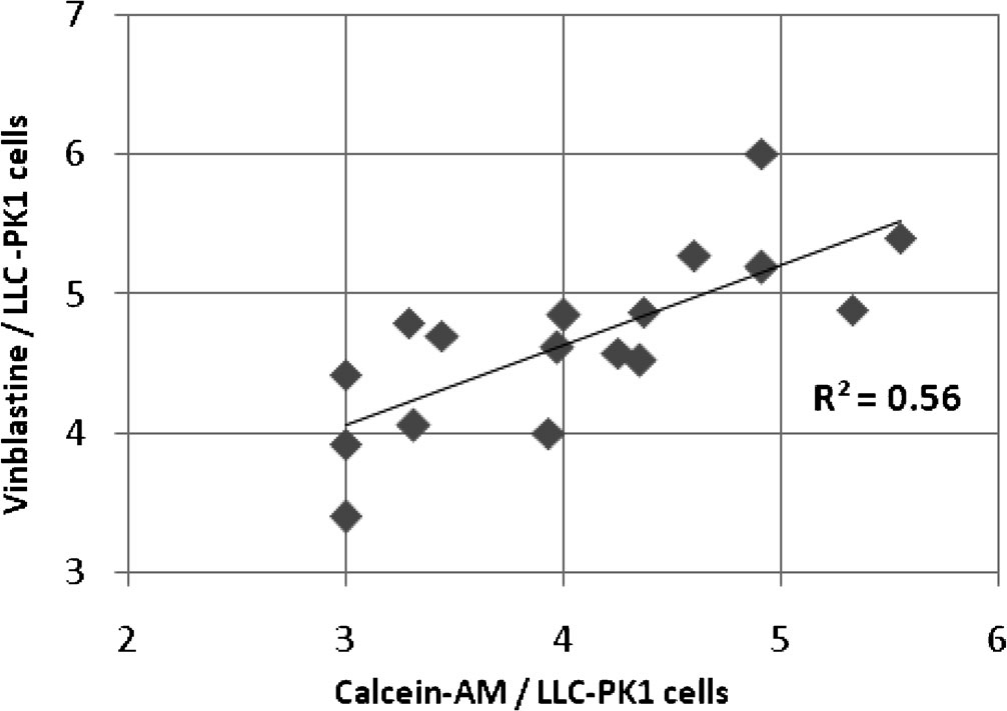
Bioactivity correlation plot: Increase in intracellular [^3^H]-vinblastine accumulation *vs.* increase in intracellular calcein-AM accumulation in LLC-PK1 cells; units in p*K*_i_.

Naturally, *K*_i_ values relate more closely to competitive inhibitors and a true binding affinity for P-glycoprotein than *IC*_50_ values. Ekins et al.^21^ published a set of 17 compounds measured in both a [^3^H]-vinblastine (radiolabeled) accumulation assay and a calcein-AM (fluorescent) accumulation assay. Both assays are of the same type (‘Increase in X intracellular accumulation’) and they were both measured in LLC-PK1 (pig kidney epithelial) cells. Such a comparison is best suited for predicting whether two different substrates have overlapping binding sites in this protein or not. In the case of vinblastine and calcein it has been proposed that their binding sites only partially overlap.^22^ That might be the reason for the rather poor correlation of the bioactivities (p*K*_i_ values; *R*^2^=0.56; Figure 3 and Table 1).

Other correlations that were obtained from ChEMBL and TP-search are all based on comparisons of p*IC*_50_ values.

**Table 1:** Bioactivities (*K*_i_ in µM; p*K*_i_) measured in same assay type (‘Increase in X intracellular accumulation’): different substrates, same cell line.

Cell line=LLC-PK1	*K*_i_ (µM), Calcein	p*K*_i_	*K*_i_ (µM), [^3^H]-Vinblastine	p*K*_i_
Bromocriptine	2.8	5.6	4.0	5.4
Clotrimazole	44.0	4.4	29.9	4.5
Cyclosporin A	4.7	5.3	1.3	4.9
Dihydro-ergocristine	511.0	3.3	16	4.8
Dihydro-ergocryptine	360.5	3.4	19.8	4.7
Dihydro-ergotamine	>1000	<3.0	119.9	3.9
Ergocornine	105.2	4.0	24.5	4.6
Ergocristine	42.8	4.4	13.3	4.9
Ergocryptine	12.2	4.9	6.4	5.2
Ergometrine	115.5	3.9	>100	4.0
Ergotamine	98.9	4.0	14.3	4.8
Erythromycin	>1000	<3.0	37.8	4.4
Fluconazole	>1000	<3.0	>400	3.4
Ketoconazole	24.9	4.6	5.3	5.3
Miconazole	55.5	4.3	26.4	4.6
Reserpine	12.2	4.9	1.0	6.0
Troleandomycin	483.3	3.3	87.6	4.1

Using a fluorescence indicator (displacement) assay (assay type: ‘Inhibition of X (transepithelial) transport’) with different fluorescence markers (=fluorescent substrates of P-glycoprotein) but always the same cell line, NIH-3T3-G185 (*MDR1* transfected mouse embryo fibroblast cell line), Wang et al.^23^ evaluated the effect of various inhibitors of P-glycoprotein on the active transport of the fluorescent substrates daunorubicin (DNR), LDS-751 (LDS), and rhodamine 123 (Rho).

**Figure 4:**
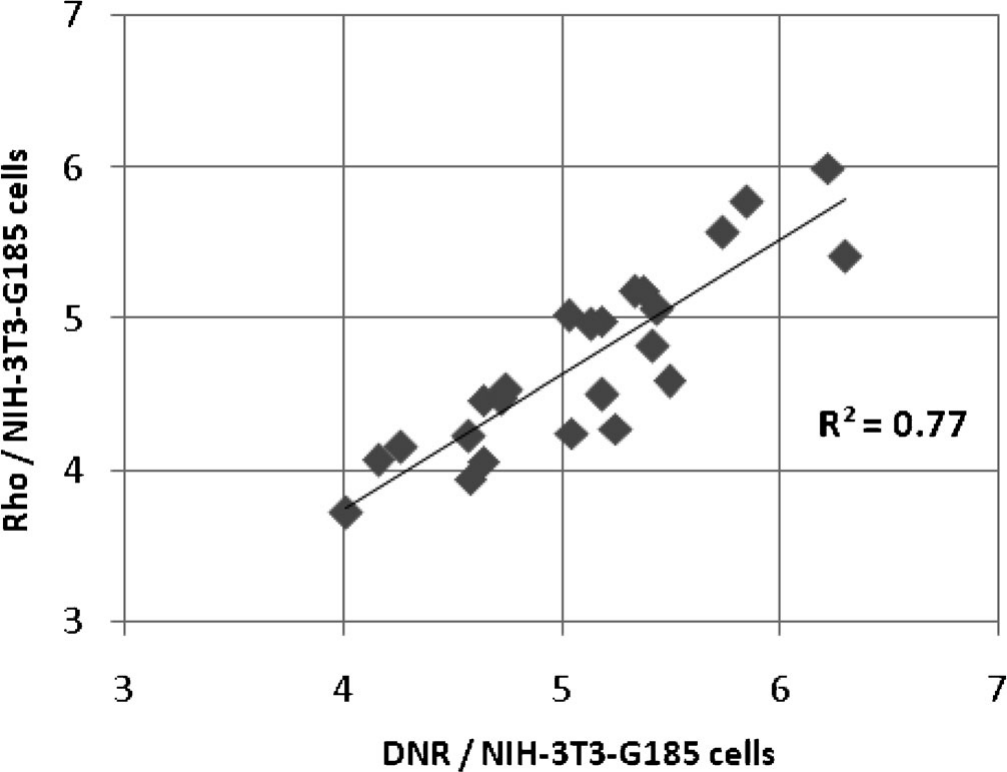
Bioactivity correlation plot: Inhibition of Rho transport vs. inhibition of DNR transport in NIH-3T3-G185 cells; units in p*IC*_50_.

**Figure 5:**
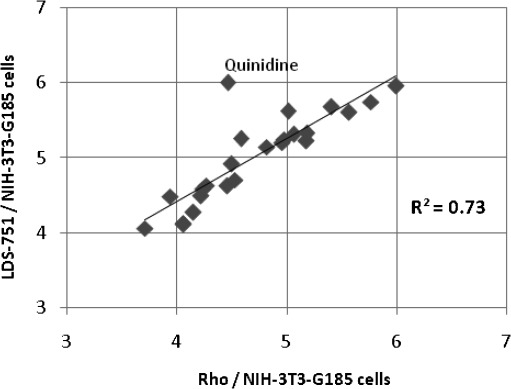
Bioactivity correlation plot: Inhibition of LDS transport vs. inhibition of Rho transport in NIH-3T3-G185 cells; units in p*IC*_50_.

**Figure 6:**
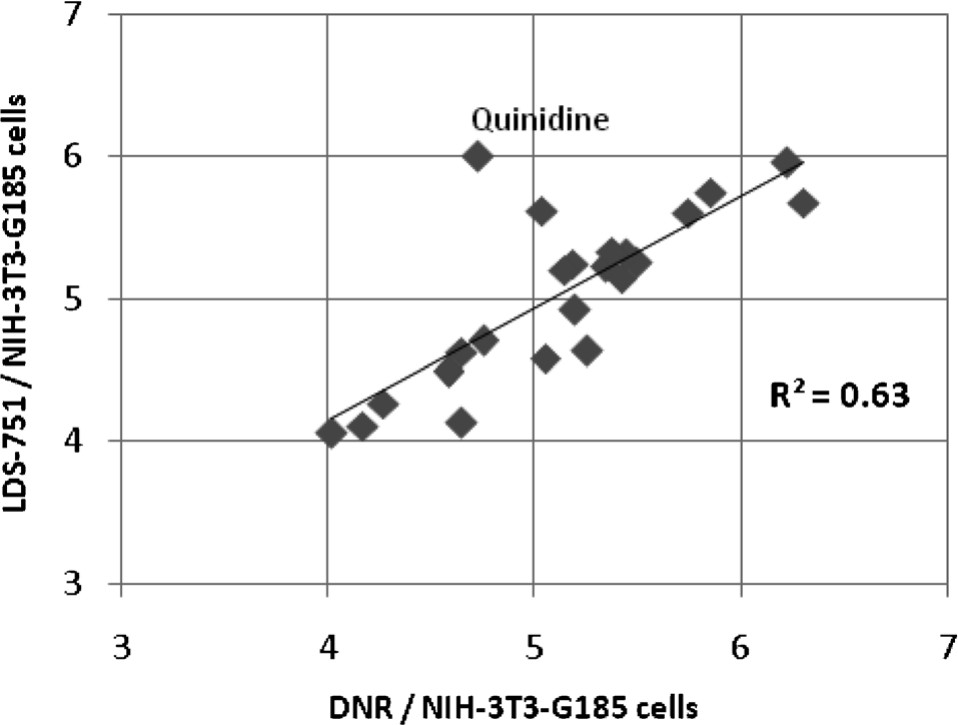
Bioactivity correlation plot: Inhibition of LDS transport vs. inhibition of DNR transport in NIH-3T3-G185 cells; units in p*IC*_50_.

The resulting activities show very similar values for most of the compounds when comparing the three different substrates (see Figures 4–Figure 6 and Table 2). Just one compound, quinidine, has a much more potent effect on LDS-751 (*IC*_50_=1.0 µM) compared to the other substrates (DNR: *IC*_50_=18.8 µM; Rho 123: *IC*_50_=33.9 µM). Also, if the bioactivity of quinidine measured with LDS-751 as marker is correct, this gives some very specific information about the LDS binding site, since its stereoisomer quinine has a 75-fold decreased affinity value. Of course, the difference in potency also affects the obtained correlation coefficients realized for the comparisons LDS-751/DNR and LDS-751/Rho (see Figures 5 and Figure 6).

Removing this outlier from the two correlations we achieved very good *R*^2^ values of 0.82 (LDS-751/DNR) and 0.90 (LDS-751/Rho), respectively. It is well known that different substrate markers favour different substrate binding sites of P-glycoprotein^24^–^26^ and that their responsiveness and wide applicability depends on their balanced affinity to more than one site.^23^ Thus, the ability to combine assays with different underlying (fluorescent or radiolabeled) substrates strongly depends on the substrates’ overlap of binding sites as well as on the series of compounds (inhibitors) under investigation.

In the cases of daunorubicin, LDS-751, and rhodamine 123 it has been postulated that all three preferentially bind to the same site (the so called ‘R site’).^25^,^27^ This was perfectly reflected by the correlations we obtained.

**Table 2:** Bioactivities (*IC*_50_ in µM; p*IC*_50_) measured in same assay type (‘Inibition of transepithelial transport’): different substrates, same cell line.

Cell line=NIH-3T3-G185	*IC*_50_ (µM), DNR	p*IC*_50_	*IC*_50_ (µM), Rho	p*IC*_50_	*IC*_50_ (µM), LDS	p*IC*_50_
Carvedilol	4.6	5.3	6.6	5.2	6	5.2
Clarithromycin	3.8	5.4	15.1	4.8	7.2	5.1
Clofazimine	0.6	6.2	1	6.0	1.1	6.0
Cyclosporin A	1.4	5.9	1.7	5.8	1.8	5.7
Dipyridamole	22.7	4.6	34.3	4.5	23.7	4.6
Emetine	9.2	5.0	9.6	5.0	2.4	5.6
Felodipine	26.3	4.6	>60	4.2	32.3	4.5
Fluphenazine	6.5	5.2	10.4	5.0	5.7	5.2
Ketoconazole	5.6	5.3	53.4	4.3	23.4	4.6
Lovastatin	26.3	4.6	114.4	3.9	32.7	4.5
Nicardipine	3.2	5.5	>25.8	4.6	5.6	5.3
N-Norgallopamil	3.6	5.4	8.6	5.1	4.9	5.3
Progesterone	96.2	4.0	192.2	3.7	88.1	4.1
Quinidine	18.8	4.7	33.9	4.5	1	6.0
Quinine	22.6	4.6	87.6	4.1	74.4	4.1
Reserpine	0.5	6.3	3.9	5.4	2.1	5.7
Simvastatin	8.9	5.1	56.8	4.2	26.1	4.6
Tamoxifen	6.4	5.2	31.4	4.5	12.1	4.9
Taxol	54	4.3	70.2	4.2	53.9	4.3
Terfenadine	1.8	5.7	2.7	5.6	2.5	5.6
Trifluo-erazine	7.2	5.1	10.9	5.0	6.3	5.2
Troleando-mycin	68.3	4.2	86.3	4.1	78.2	4.1
Verapamil	4.2	5.4	6.5	5.2	4.7	5.3
Vinblastine	17.7	4.8	29.5	4.5	19.9	4.7

#### 3.3.2 Comparison: Same Assay Type, Same Substrate (X), Different Cell Lines (Expressing P-Glycoprotein from Different Species)

**Figure 7:**
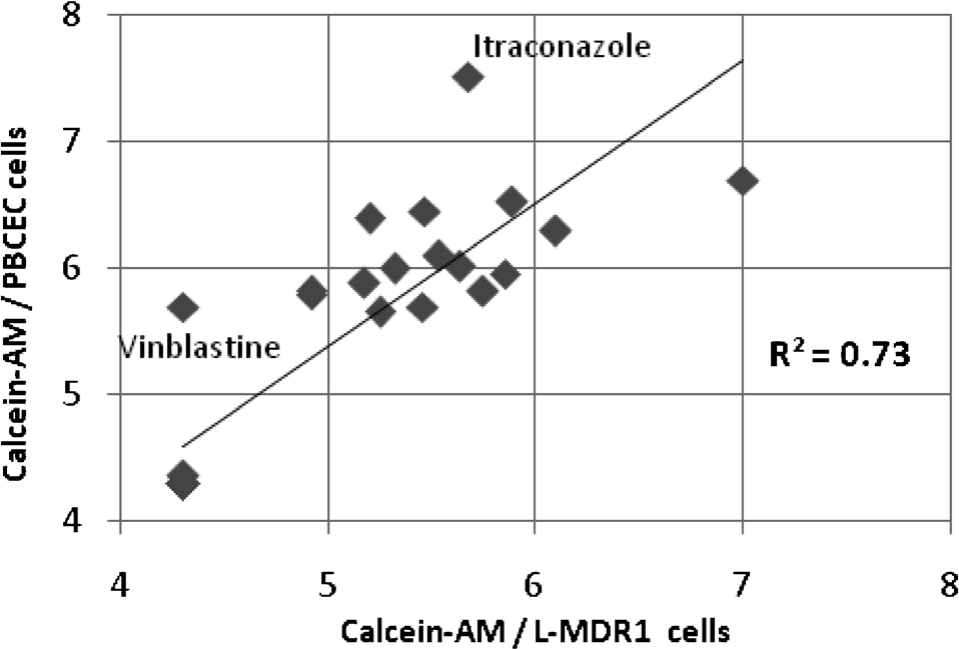
Bioactivity correlation plot: Increase in intracellular calcein accumulation: measured in PBCEC cells (porcine) vs. L-MDR1 cells (human); units in p*IC*_50_.

The question as to whether identical assay setups, but different cell lines, will lead to similar activity values becomes even more interesting if cell lines under investigation are expressing P-glycoprotein from different species. Schwab et al.^28^ measured in an indirect indicator assay with Calcein-AM as fluorescent dye (assays type: ‘Increase in X intracellular accumulation’) the P-glycoprotein inhibitory activity of 28 compounds in both polarized pig kidney epithelial LLC-PK1 cells transfected with human MDR1 (L-MDR1 cells) and in porcine brain capillary endothelial cells (PBCEC cells). There was quite a good correlation between the p*IC*_50_ values obtained using cells expressing human or porcine protein (*R*^2^=0.73; Figure 7 and Table 3). Vinblastine was differently classified in both cell lines, being an inhibitor in PBCEC cells but not in L-MDR1 cells. On removal of this outlier from the correlation plot an *R*^2^ of 0.78 could be achieved. As a general tendency of this correlation we observed that a few inhibitors had lower *IC*_50_ values in PBCEC cells (itraconazole, ritonavir, saquinavir, verapamil). Particularly noticeable is itraconazole with a 70-fold higher inhibitory activity measured in porcine cells than in those transfected with human P-glycoprotein (*R*^2^ [vinblastine and itraconazole removed] =0.83).

By performing such comparisons (between P-glycoprotein from different species), outliers might provide some species specific information about binding to this protein.

In general, combining assay data from different cell lines is quite risky, as distinct types of cells might also express other ABC-transporters with compound binding profiles partly overlapping with those for P-glycoprotein, rendering data interpretation even more difficult.

**Table 3:** Bioactivities (*IC*_50_ in µM; p*IC*_50_) measured in same assay type (‘Increase in X intracellular accumulation’): same substrate, different cell lines.

Calcein accumulation/cell line:	*IC*_50_ (µM), L-MDR1	p*IC*_50_	*IC*_50_ (µM), PBCEC	p*IC*_50_
Astemizole	1.30	5.9	0.30	6.5
Cimetidine	>50	4.3	>50	4.3
Clotrimazole	6.70	5.2	1.30	5.9
Colchicine	>50	4.3	>50	4.3
Cyclosporin A	0.80	6.1	0.50	6.3
Dexamethasone	>50	4.3	>50	4.3
Digoxin	>50	4.3	>50	4.3
Enkephalin (dpdpe)	>50	4.3	>50	4.3
Erythromycin	>50	4.3	43.00	4.4
Etoposide	>50	4.3	>50	4.3
Hydrocortisone	>50	4.3	>50	4.3
Ivermectin	0.10	7.0	0.20	6.7
Ketoconazole	4.8	5.3	1.00	6.0
Mibefradil	1.80	5.7	1.50	5.8
Miconazole	3.50	5.5	2.00	5.7
Midazolam	>50	4.3	>50	4.3
Morphin	>50	4.3	>50	4.3
Nelfinavir	3.40	5.5	0.35	6.5
Nicardipine	2.3	5.6	0.95	6.0
Pimozide	2.90	5.5	0.80	6.1
Quinidine	5.6	5.3	2.20	5.7
Ranitidine	>50	4.3	>50	4.3
Ritonavir	12.00	4.9	1.50	5.8
Saquinavir	12.00	4.9	1.60	5.8
Terfenadine	1.4	5.9	1.10	6.0
Verapamil	6.3	5.2	0.40	6.4
Vinblastine	>50	4.3	2.00	5.7

#### 3.3.3 Comparing Different Assays Types

**Figure 8:**
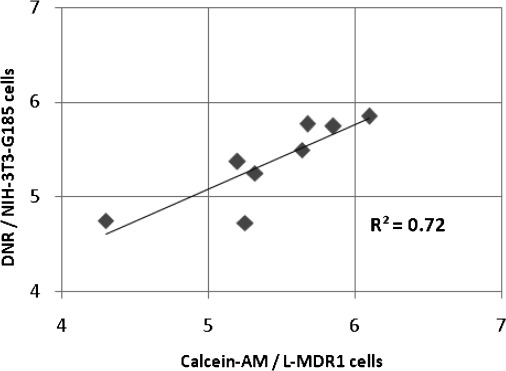
Bioactivity correlation plot: Increase in DNR accumulation in L-MDR1 cells vs. increase in calcein-AM accumulation in NIH-3T3-G185 cells; units in p*IC*_50_.

Going further with the analysis, we also tried to combine data from different assay types, with different marker (substrate) and different cell lines. Because it is very probable that different labs performing the same assay might also achieve slightly different results, we established correlations by taking into account only data from one publication for each test series. Unfortunately, this led to data sets with an overlap of only 7–8 compounds, which is probably not very representative. Still, we found a good correlation (*R*^2^=0.72; Figure 8 and Table 4) comparing the p*IC*_50_ values of a calcein-AM accumulation assay in L-MDR1 cells to those of a daunorubicin transport assay in NIH-3T3-G185 cells. However, when comparing results from a calcein-AM/L-MDR1 accumulation assay to those of transport assays with rhodamine 123/NIH-3T3-G185 (*R*^2^=0.4) or with LDS-751/NIH-3T3-G185 (*R*^2^=0.36), we were not able to establish any correlations between these combinations of substrates and cell lines (data not shown). Data extracted from ChEMBL/TP-search were originally reported in the publications of Schwab et al.^28^ and Wang et al.^23^ (as described earlier).

**Table 4:** Bioactivities (*IC*_50_ in µM; p*IC*_50_) measured in same assay type (‘Increase in X intracellular accumulation’): different substrate, different cell lines.

	*IC*_50_ (µM), Calcein/L-MDR1	p*IC*_50_	*IC*_50_(µM), DNR/NIH-3T3-G185	p*IC*_50_
Cyclosporin A	0.8	6.1	1.4	5.9
Itraconazole	2.1	5.7	1.7	5.8
Ketoconazole	4.8	5.3	5.6	5.3
Nicardipine	2.3	5.6	3.2	5.5
Quinidine	5.6	5.3	18.8	4.7
Terfenadine	1.4	5.9	1.8	5.7
Verapamil	6.3	5.2	4.2	5.4
Vinblastine	>50	4.3	17.7	4.8

Secondly, we also studied possible correlations for a series of tetrahydroisoquinoline and piperazine derivatives measured in a calcein-AM accumulation assay in MDCK-MDR1 cells versus a [^3^H]-vinblastine transport inhibition assay with Caco-2 cells. An *R*^2^ of 0.07 indicated no correlation at all between these assays (data not shown). The authors of this study (Colabufo et al.)^29^ argued that this lack of any correlation could be either due to the different cell lines or to the different underlying assays (with different sensitivities).^26^ The result is also in accordance with the study by Ekins et al.^21^ which suggests that vinblastine and calcein might only have partially overlapping binding sites. We also observed that correlations were better for one compound class (tetrahydroisoquinolines) than for the other (piperazines). That points to the influence of the inhibitors’ chemical structure on the usability of certain bioassays.

More broadly, this analysis implies that the prediction of drug-drug interactions will require both a better characterisation of existing drugs and development of sub-site specific models in order to have higher predictivity.

### 3.4 QSAR and Classification Datasets for Human P-Glycoprotein Inhibitors

Based on this study it was not possible to build up a large dataset of P-glycoprotein inhibitors based on the combination of different assays. The largest set contains 198 compounds and was obtained by merging three SAR datasets from ChEMBL, which were all measured in a daunorubicin transport assay in MDR CCRF vcr1000 cells (see Supporting Information, File S1).^18^–^20^ Even though 24 % of the compounds with *IC*_50_ values for human P-glycoprotein in TP-search have also been measured in a daunorubicin transport assay (see Figure 2), none of these assays uses CCRF vcr1000 cells. Our studies suggest that bioactivities from daunorubicin-, LDS-751-, and rhodamine 123-transport assays can be combined in the case of NIH-3T3-G185 cells. It is not clear if the same is true for other cell lines. Thus, it was not possible to combine the 198 dataset with other datasets measured in different assay setups.

Recently, other groups combined different literature sources (and thus data coming from different assays) for the generation of large classification databases by setting certain thresholds.

Broccatelli et al.^30^ used a classification scheme derived from the observations of Rautio et al.^22^ inhibitor (*IC*_50_)<15 µM; non-inhibitor (*IC*_50_)>100 µM. Rautio et al. tested twenty compounds with different protocols using five different probe substrates. However, not all combinations of assay types/probe substrates/cell lines could be taken into account.

Chen et al.^31^ based the determination of whether a compound is an inhibitor or not on the experimental MDRR (multidrug resistance reversal) ratio. Comparing these two databases (of approximately 1200 compounds each) we found 33 collisions (differently classified compounds).

From our point of view, one can achieve a cleaner and more robust dataset by not considering only one threshold, but by using a tailored threshold for every assay. This could be fulfilled by carefully inspecting the bioactivity measures of a compound that has been determined in many different assays. To our knowledge, verapamil is one of the broadest studied molecules interacting with P-glycoprotein. Table 5 lists all the *IC*_50_ values for verapamil with the respective underlying assays that we could find in ChEMBL and TP-search for human P-glycoprotein. Considering the respective (inhibitor) assays with determined verapamil-activity, we could classify all compounds having an equal or better affinity/potency than verapamil in a certain assay as inhibitor. Compounds with an activity value lower than verapamil were classified as non-inhibitors. In this way we created a benchmark dataset useful for P-glycoprotein classification studies, which comprises 77 inhibitors and 126 non-inhibitors (see Supporting Information, File S2).

We recommend measuring verapamil activity routinely when running a certain assay for a compound dataset. As a consequence, the pool of available information on verapamil thresholds will increase and then further serve for building up larger classification databases. In addition, verapamil bioactivities in certain assay setups already give an indication of the ability to combine them.

**Table 5:** *IC*_50_ values (in µM) for verapamil in ChEMBL and TP-search.

Assay	Cell line	Substrate (=X)	*IC*_50_ (μM)
Inhibition of X transepithelial transport	Caco-2	Digoxin	2.1
	Caco-2	Fexofenadine	8.4
	LLC-PK1	Digoxin	224.0
	LLC-PK1	Calcein-AM	6.3
	NIH-3T3-G185	DNR	4.2
	NIH-3T3-G185	Rho123	6.5
	NIH-3T3-G185	LDS-751	4.7
	NIH-3T3-G185	Fluo-3-AM	446.5
	NIH-3T3-G185	Calcein-AM	28.9
	NIH-3T3-G185	JC-1	42
	NIH-3T3-G185	Tetramethyl-rosamine	38.2
Increase in X intracellular accumulation	MDCK	Rho123	9.8
	EMT6/AR 1.0	DNR	5.8
	MDCK2	Calcein-AM	14.0
	PBCEC	Calcein-AM	0.4
	LLC-PK1	Calcein-AM	6.3
	L-MDR1	Calcein-AM	18.9
Reversal of MDR	P388/VMDRC.04	Vincristine	3.1
	AML-2/D100	Vincristine	0.4
	MES-SA/DX5	Paclitaxel	4.8
	MES-SA/DX5	Paclitaxel	5.3^[b]^
	MES-SA/DX5	Paclitaxel	5.5^[a]^
	HCT15/CL02	Paclitaxel	2.2
	HCT15/CL02	Paclitaxel	2.4^[b]^
	HCT15/CL02	Paclitaxel	2.6^[a]^
Cytotoxic effect	P388/VMDRC.04	x	53.0
Radioligand-binding assay (competition experiment)	Caco-2	[^3^H]-verapamil	1.5
	Caco-2	[^3^H]-verapamil	2.4^[a]^
	Caco-2	[^3^H]-verapamil	2.1

[a] R-Enantiomer; [b] S-Enantiomer.

## 4 Conclusions

In conclusion, combining different bioactivity values from different assay setups should always be made with caution. Our study indicates that for inhibitors of human P-glycoprotein it is possible under certain conditions to combine data coming from the same assay type, if the cell lines used are also identical and the fluorescent or radiolabeled substrate have overlapping binding sites.

As a result of this study we have determined that there is only a very limited number of datasets with an appropriate number of compounds (at least ten) available, which have been measured in more than one assay and thus can serve for determining correlations. In order to prove our hypotheses, and to be able to find new inter-correlations between assays, there is an urgent need for a chemically diverse dataset measured in a panel of different assays, with different markers, and different cell lines.

It was observed throughout this study that expert knowledge is needed to organize and annotate existing bioactivity data, in this case specifically for human P-glycoprotein. There are several useful tools available which could help to increase the systematic structuring of bioassay data. One is MIABE (Minimum Information About a Bioactive Entity), which provides a formal list of information that should be provided when describing the synthesis and subsequent analysis of any potentially bioactive entity.^32^ Another example is the STRENDA (standards for reporting enzymological data)^33^ Commission. Again, this initiative aims at providing a check-list of information that should be included when reporting data, but focusing on enzyme data. In addition, STRENDA tends to give recommendations for uniform assay standards and standardization of enzyme data. Above all, the BioAssay Ontology project (BAO) should be mentioned, which has been initiated to facilitate the standardization of annotating the screening setup and the data generated.^34^ In the BAO, there are already more than 350 assays from PubChem annotated with standardized BAO terms.^35^ By defining the assay type and the compound action, functional viability and uptake assays can be separated from binding assays, while the activation or inhibition is annotated elsewhere. A standardized vocabulary enables extended searches regarding assay design, which can include both detection technology and instrument, dye specification, and other assay conditions. However, of the ABC superfamily of transport proteins there is only one member, the Multidrug resistance-associated protein 1 (UniProt ID: P33527; alternative protein names: ATP-binding cassette sub-family C member 1, Leukotriene C(4) transporter=LTC4 transporter; gene names: ABCC1, MRP, or MRP1) represented in the beta version of the BAO search tool.^36^ This indicates that by far more work needs to be done to improve the coverage of this important antitarget family. Emerging standardization efforts as represented by BAO also underpin the importance of a bioassay annotation/classification at the time of assay data disposition.

Standardized vocabulary for assay design and technology would also allow making data interoperable and would remarkably increase the capabilities of data integration platforms, such as Open PHACTS.^37^,^38^ This furthermore will promote enhanced access and use of data within both public sources and the pharmaceutical industry. Even more interesting would be the possibility to mark specific assays as interchangeable, which would allow to remarkably amplify the chemical space for certain targets. This will significantly increase the usefulness of those huge data repositories freely available.

## Supporting Information

**Table S1**: Regression statistics of correlation plots (Figures 3–8). **Table S2**: Cell lines used for bioassays determining bioactivities of compounds interacting with human P-glycoprotein in ChEMBL and TP-search. **File S1**: sdf file of 198 chemical compounds with chemical structures and bioactivity values included measured in a daunorubicin efflux assay in MDR CCRF vcr1000 cells. **File S2**: sdf file of 203 chemical compounds being either classified as inhibitors or non-inhibitors of human P-glycoprotein with chemical structures included.

In addition, sdf-files of the two datasets will also be available from our web-page pharminfo.univie.ac.at and from chemspider.com.
