# Supplementary material for: Annotating Human P-Glycoprotein Bioassay Data
Source: Mol Inform. 2012 Aug 7;31(8):599–609. doi: 10.1002/minf.201200059 (PMC3531886; doi:10.1002/minf.201200059)
Supplement: Supplementary file 1 [file minf0031-0599-sd1.pdf]

molecular informatics

models – molecules – systems

Supporting Information

© Copyright Wiley-VCH Verlag GmbH & Co. KGaA, 69451 Weinheim, 2012

Supporting Information.

Table S1.

Regression statistics of correlation plots (Figures 3-8).

**Regression statistics**<br>**Confidence level = 95%**	**Fig. 3**	**Fig. 4**	**Fig. 5**	**Fig. 6**	**Fig. 7**	**Fig. 8**
Correlation Coefficient (R)	0.748	0.879	0.855	0.791	0.855	0.847
Coefficient of Determination (R^2)	0.560	0.772	0.732	0.626	0.731	0.717
Adjusted R^2	0.530	0.762	0.719	0.609	0.721	0.669
Standard Error	0.427	0.295	0.316	0.372	0.519	0.253
Observations	17	24	24	24	28	8

Table S2.

Cell lines used for bioassays determining bioactivities of compounds interacting with human P-glycoprotein in ChEMBL and TP-search.

**Cell line**	**Description**	**Species**
AML-2/D100	daunorubicin-resistant acute myelogenous leukemia cell line	human
BHK	baby hamster kidney cell line	hamster
Caco-2	human epithelial colorectal adenocarcinoma cells	human
CCRF/VCR1000	human T lymphoblastoid cell line	human
CEM	human leukemic cell line	human
CEM/VLB100	vinblastine-resistant T-lymphoblastic leukaemic cell line	human
HCT15/CL02	doxorubicin-resistant subline from parental HCT15 human adenocarcinoma colon cancer cells	human
HCT-8	human colon cancer cell line	human
HEK	human embryonic kidney 293 cell line	human
HL60/Vinc	vincristine-resistant HL60 human promyelocytic leukaemia cell line	human
HL60R	multi-drug resistant human promyelocytic leukemia cell line	human
K562/Adr	adriamycin-resistant human erythroleukemia cell line	human
KB/MDR	human nasopharyngeal carcinoma KB cell line	human
KB-V1	multidrug-resistant cervical cancer cell line	human
L5178Y	murine lymphoblastic cell lines	mouse
LLC-PK1	pig kidney epithelial cells	pig
L-MDR1	polarized LLC-PK1 epithelial cells transfected with MDR1	human
MDA435/LCC6/MDR1	P-gp overexpressing human breast cancer cell line	human
MDCK	Madin-Darby canine kidney cell line	dog
MDCK2	cell line derived from MDCK	dog
MDR-CEM	multi-drug resistant (MDR) variant of the CEM human T lymphoblastoid cell line	human
MDR-P388	multi-drug resistant subline of murine monocytic leukemia P388 cell line	mouse
MES-SA/DX5	multi-drug resistant human uterine sarcoma cell line	human
MRP1-transfected HeLa-T5 cell line	mutidrug resitant human cervical cancer cell line	human
NIH-3T3-G185	MDR1 transfected mouse embryo fibroblast cell line	mouse
P388/VMDRC.04	multi-drug resistant subline of P388 murine leukemia cells	mouse
PBCEC	porcine brain capillary endothelial cell line	pig

File S1.: “QSAR_dataset_198cpds.sdf”

sdf file of 199 chemical compounds with chemical structures and bioactivity values included measured in a daunorubicin efflux assay in MDR CCRF vcr1000 cells.

File S2.: “Classification_dataset_203cpds.sdf”

sdf file of 203 chemical compounds being either classified as inhibitors or non-inhibitors of human P-glycoprotein with chemical structures included.
